# Toward a regional digital pathology network in Tuscany: current status and implementation roadmap

**DOI:** 10.1007/s00428-026-04408-2

**Published:** 2026-01-28

**Authors:** Giuseppe Nicolò Fanelli, Filippo Ugolini, Alessandro Ginori, Anna Maria Buccoliero, Alessandra Calcinai, Andrea Carnevali, Angelo Cassisa, Barbara Cappelli, Luisa Cattaneo, Davide Cerratani, Nicola Libertà Decarli, Giulia Garavello, Stefano Lazzi, Vincenzo Nardini, Gabriella Nesi, Raffaella Santi, Cristian Scatena, Clara Ugolini, Paolo Viacava, Antonio Giuseppe Naccarato, Daniela Massi

**Affiliations:** 1 Division of Pathology, Department of Translational Research and New Technologies in Medicine and Surgery, University of Pisa, Pisa, Italy; 2 First Division of Pathology, Department of Oncology, Pisa University Hospital, Pisa, Italy; 3 Department of Pathology and Laboratory Medicine, Weill Cornell Medical College, New York, NY USA; 4 Unit of Histopathology and Molecular Diagnostics, Careggi University Hospital, Florence, Italy; 5 Pathology Unit, Azienda USL Toscana Nord-Ovest, Carrara Hospital, Carrara, Italy; 6 Pathology Unit, Meyer Childrenʼs Hospital IRCCS, Florence, Italy; 7 Pathology Unit, USL Toscana Centro, Pescia Hospital, Pescia, Italy; 8 Pathology Unit, Oncology Department, San Donato Hospital, Arezzo, Italy; 9 Section of Pathology, Department of Oncology, San Giovanni Di Dio Hospital, USL Toscana Centro, Florence, Italy; 10 ICT Department, ESTAR, Florence, Italy; 11 Pathology Unit, Misericordia Hospital, Grosseto, Italy; 12 Pathology Unit, USL Toscana Centro, Empoli Hospital, Empoli, Italy; 13 Institute of Pathology, Department of Medical Biotechnology, Siena University Hospital, Siena, Italy; 14 Second Division of Pathology, Department of Oncology, Pisa University Hospital, Pisa, Italy; 15 Section of Anatomic Pathology, Department of Health Sciences, University of Florence, Florence, Italy; 16 Third Division of Pathology, Department of Oncology, Pisa University Hospital, Pisa, Italy; 17 Division of Pathology, Department of Surgical, Medical, and Molecular Pathology, University of Pisa, Pisa, Italy; 18 Pathology Unit, Azienda USL Toscana Nord-Ovest, Livorno Hospital, Livorno, Italy; 19 Department of Molecular Pathobiology, New York University - College of Dentistry, New York, NY USA

**Keywords:** Digital pathology, Telepathology, Artificial intelligence, Regional healthcare system, Precision medicine, Workflow integration

## Abstract

Digital pathology (DP) is reshaping diagnostic workflows, offering enhanced accuracy, efficiency, and collaboration through high-resolution slide scanning, artificial intelligence (AI), and cloud-based infrastructure. In Tuscany, the adoption of DP is framed within a regionally integrated healthcare system composed of three local health authorities and four university hospitals, coordinated under a hub-and-spoke model. This structure supports the potential for widespread DP implementation, leveraging centralized expertise and shared digital infrastructure. A region-wide survey involving all public pathology centers in Tuscany confirmed that all institutions are already equipped with at least one whole slide scanner. Building on this foundation, the region has initiated a strategic transformation stepwise process to implement and progressively expand DP workflows. Key actions included the adoption of a common regional laboratory information system (LIS), the development of a centralized cloud repository ensuring secure data access, and the design of telepathology modules to enable remote consultations and second opinions. Dedicated training programs for technical staff and the progressive introduction of AI-assisted tools are also part of this roadmap, ensuring readiness for routine clinical integration. The benefits of DP in Tuscany are manifold: faster diagnostic turnaround, improved inter-institutional collaboration, standardized reporting, and opportunities for research and education. Integration of AI-assisted tools is expected to support routine diagnostics, especially in high-volume and complex cases. The regional network also creates a foundation for multi-omics integration and computational pathology research. To ensure successful implementation, the region adopted a phased, scalable approach backed by regulatory alignment and continuous professional development. A unified DP network with shared protocols and centralized resources will be crucial. Tuscany’s experience may serve as a blueprint for other regions aiming to transition toward a digital, AI-powered pathology ecosystem aligned with the broader goals of precision medicine.

**Supplementary Information:**

The online version contains supplementary material available at https://doi.org/10.1007/s00428-026-04408-2.

## Introduction

The growing demand for precision medicine, driven by the increasing complexity of cancer diagnostics and targeted therapies, has placed new burdens on pathology. Modern oncology relies on molecular profiling, immunohistochemistry, histopathological classifications, and grading/staging systems, all of which require high levels of accuracy and reproducibility [1, 2]. For over a century, surgical pathology has been based on glass slides and microscopes; however, advances in high-resolution imaging, cloud computing, and artificial intelligence (AI) have catalyzed the rise of digital pathology (DP) as a transformative force in diagnostics [3]. DP involves the acquisition, management, analysis, and interpretation of pathology data in a digital framework, primarily leveraging whole slide imaging (WSI) to convert glass slides into high-resolution digital images that can be viewed, shared, and analyzed using dedicated platforms [4].

Pathologists worldwide face growing workloads due to personnel shortages, rising case volumes, and increasingly detailed reporting requirements [5]. In 2022, SIAPeC-IAP (the Italian Society of Anatomic Pathology and Cytology) estimated that in Italy, there are ~ 1100 practicing pathologists, a ~ 25% decrease over the past 5 years [6], with many nearing retirement and fewer residents in training. Meanwhile, the *2024 Italian Cancer Numbers Report* recorded ~ 390,000 new cancer diagnoses, with incidence projected to rise by ~ 20% in 2040 [7]. This imbalance between workforce contraction and growing demand further intensifies the diagnostic burden on pathology services. DP offers a potential solution to relieve this challenge, providing several advantages, including (i) *efficiency gains*: faster turnaround times (TAT) and remote access to slides, reducing diagnostic delays and improving patient management [3]; (ii) *improved reproducibility*: standardized digital workflows reduce interobserver variability, ensuring consistent and reliable diagnoses and supporting large‑scale research collaborations [8, 9]; (iii) *pathologist shortage mitigation*: AI-powered pre-screening allows pathologists to focus on cases requiring human expertise [10, 11], while cloud-based storage and telepathology facilitate remote collaboration, particularly in understaffed regions/services [12–15]; (iv) *AI integration*: AI-models integration opens new opportunities in diagnostics, enhancing accuracy and efficiency [12, 13, 16–18].

Meanwhile, the regulatory landscape is evolving to harmonize and govern these innovations, ensuring compliance with GDPR and national policies [19–22]. Notably, DP solutions fall under the definition of in vitro diagnostic medical devices (IVDs) when the software is “specifically intended by the manufacturer to be used for one or more medical purposes listed in the definition of an IVD” as established by Regulation (EU) 2017/746 (IVDR). Such classification requires compliance with CE marking procedures, performance evaluation, clinical validation, and post-market surveillance before routine clinical adoption.

The potential benefits of DP are clear, but successful implementation requires careful planning, investment, validation, regulatory approval, ongoing monitoring, and training programs for pathologists and laboratory technicians [19, 20, 23]. Addressing all these challenges will be crucial to ensuring a smooth transition from traditional histopathology to fully integrated digital workflows. These considerations have directly informed the progressive implementation of DP across the Tuscany Regional Healthcare System (TRHS), which aims to build a unified, interoperable, and scalable diagnostic network aligned with the principles of precision medicine.

**Fig. 1:**
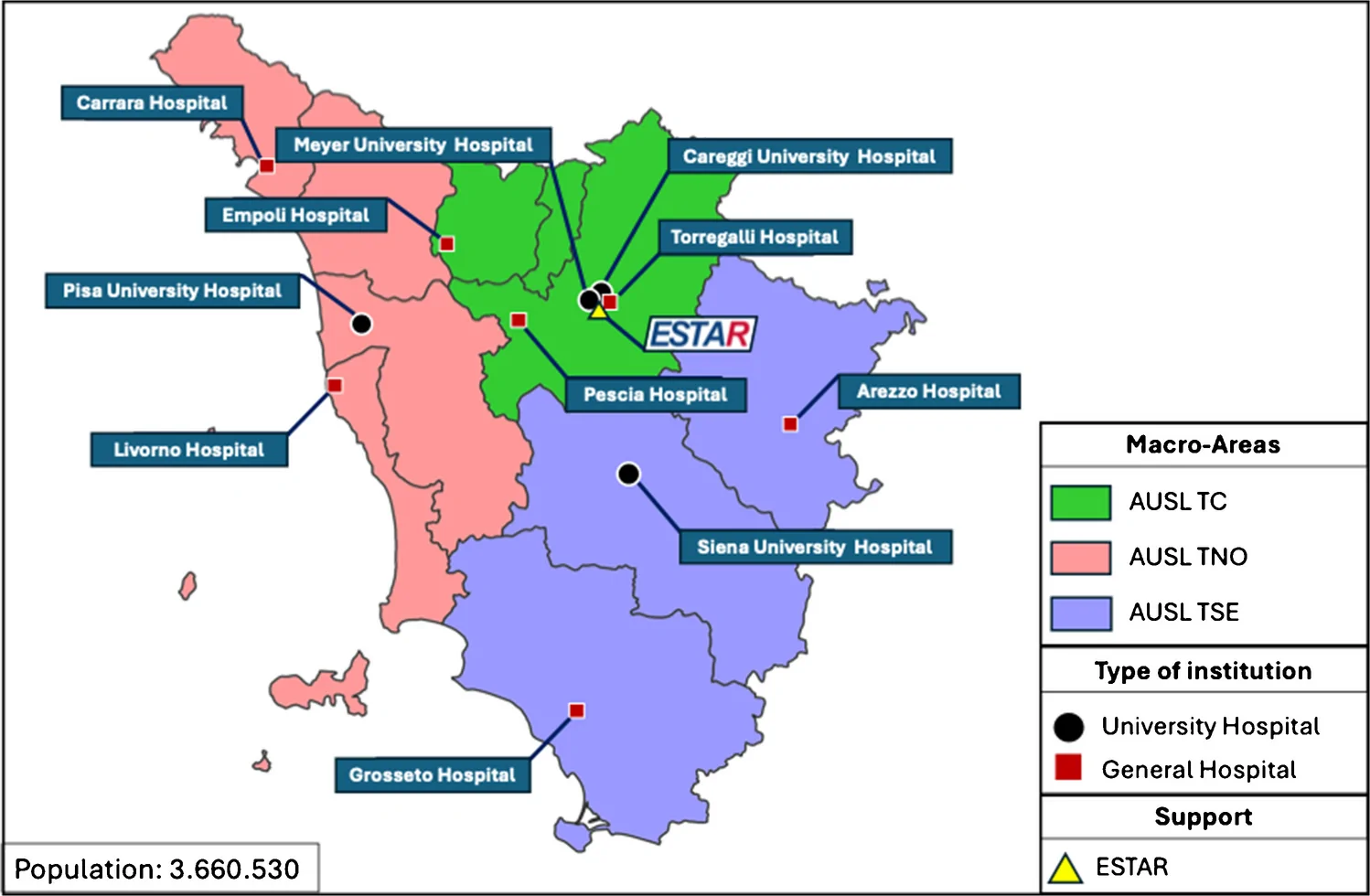
Geographic distribution of all public hospitals in Tuscany with active pathology laboratories. The map displays all hospitals across the three regional macro-areas—AUSL TC, AUSL TNO, and AUSL TSE—that currently host a pathology service. Institutions are classified as general hospitals or university hospitals. The yellow triangle indicates the location of ESTAR, the regional agency coordinating IT services and leading the implementation of the Digital Pathology program. The network serves a total population of approximately 3.66 million. AUSL, Local Health Authorities; TC, Center Area; TNO, Northwest Area; TSE, Southeast Area

The TRHS is designed as an integrated network of services aimed at ensuring comprehensive, equitable, and standardized diagnostic and therapeutic support across the entire region [24]. Territorial healthcare delivery is organized through three local health authorities (AUSL): (i) *Azienda USL Toscana Centro* (AUSL TC), covering the provinces of Empoli, Florence, Pistoia, and Prato; (ii) *Azienda USL Toscana Nord-Ovest* (AUSL TNO), serving the areas of Livorno, Lucca, Massa-Carrara, Pisa, and Viareggio; (iii) *Azienda USL Toscana Sud-Est* (AUSL SE), overseeing the provinces of Arezzo, Grosseto, and Siena. The three AUSL are organized into corresponding macro-areas, each linked with a University Hospital (Azienda Ospedaliero-Universitaria: AOU) that serves as its primary hub center: Careggi University Hospital (AOUC) for the central area, Siena University Hospital (AOUS) for the southeast, and Pisa University Hospital (AOUP) for the northwest. In addition, the Meyer Children’s Hospital (AOUM) in Florence serves as the regional pediatric reference center. To support this arrangement, the region relies on ESTAR (Ente di Supporto Tecnico-Amministrativo della Regione Toscana), the regional agency established to centralize and optimize technical, administrative, and digital services, as well as the management of biomedical and clinical systems, across TRHS. Notably, only 11 of the 43 public hospitals in the regional network currently host an in-house pathology laboratory (Fig. 1).

In this context, DP implementation is a key component of a broader strategy to modernize regional healthcare infrastructure and optimize pathology services, aligning with global trends in precision medicine and offering improved efficiency, diagnostic reproducibility, and accessibility [25]. Financial aspects are crucial: although initial investments in scanners, software, and IT infrastructure are substantial, several analyses have demonstrated long-term cost-effectiveness [26–28], with quantitative results showing that DP can achieve cost recovery within 6 years [29]. Moreover, centralized DP networks can optimize resource utilization across multiple hospitals, streamlining diagnostics and facilitating large-scale research initiatives. In Tuscany, the DP program has been fully financed, specifically for software infrastructure, through Next Generation EU funds under Italy’s National Recovery and Resilience Plan (PNRR), Mission 6 (Health), Component 2 (Innovation, research, and digitalization of the national healthcare service), Investment 1.1 (Modernization of hospital technological and digital infrastructure), Sub-investment 1.1.1 (Digitalization of Level I and II Emergency and Admission Departments—DEA), allocating € 82.4 million in Tuscany (~ € 3 million dedicated for the DP project) equally distributed across 19 main Tuscany hospitals coordinated by ESTAR [30, 31], which acts as a strategic partner by overseeing the acquisition and deployment of WSI platforms, ensuring interoperability with laboratory information systems (LIS), and enabling a cohesive digital architecture across regional hospitals. At this stage, detailed financial figures on the distribution of hardware and software investments are not yet available, as the allocation processes are still ongoing and subject to regional administrative procedures; however, the regional plan will ensure balanced allocation of resources across all centers, covering network infrastructure, software integration, maintenance, and training in a harmonized framework. A long-term performance and sustainability assessment, including evaluation of return on investment, will be conducted after full implementation. This regional plan aligns with broader European experiences in DP networking, including the participation of University of Florence/AOU Careggi in the MELCAYA project funded by the Horizon Europe Programme (Grant Agreement No. 101096667) [32]. This partnership provides a strategic advantage for the region, offering early experience in cross-institutional consultation workflows, interoperability testing, and data governance at the European level.

Here, we examine the driving forces behind the adoption of DP in Tuscany, assess its current state, and explore barriers and potential future strategies for its widespread implementation. By addressing these factors, we—healthcare policymakers, pathologists, and IT specialists—developed a sustainable roadmap for DP transformation.

## Strategic roadmap and recommendations

The implementation of DP within the TRHS represents a transformative opportunity that requires a structured, phased roadmap balancing immediate clinical needs with long-term innovation goals. It is not merely a technological shift but a comprehensive reorganization of clinical workflows, educational practices, and regulatory frameworks, ultimately aimed at improving patient outcomes [8, 10].

The first step is to complete the regional scale-up of DP by ensuring full adoption across all hospitals. This demands the standardization of imaging platforms, slide formats, and workflow protocols, as well as the consolidation of IT infrastructure. A unified technological framework will enable seamless integration of data, improve interoperability, and allow smaller hospitals to connect with academic hubs for diagnostic support, optimizing the distribution of subspecialist expertise and reducing inter-institutional disparities.

Once this infrastructure is in place, the next priority is the integration of advanced tools, such as AI and telepathology. These technologies hold immense potential for enhancing diagnostic performance, accelerating workflows, and supporting remote consultation. Their successful adoption, however, depends on robust infrastructure, validated datasets, and secure, cloud-based platforms.

Equally important is the human component of this transformation. Continuous education and training must be guaranteed for pathologists, technicians, and IT staff, ensuring digital literacy and building trust in the new tools. A stepwise rollout can support gradual adoption while minimizing resistance.

To guarantee legal compliance and data security, regulatory frameworks must evolve in parallel with technological advances. Clear guidelines are needed for digital slide retention, AI validation, access control, and data governance. Tuscany’s approach—designed around GDPR-compliant standards and built-in cybersecurity—is aligned with these priorities and can serve as a model for national policy development.

In parallel, the region should capitalize on its digital infrastructure to foster a robust research and innovation ecosystem. By connecting hospitals, universities, and industry partners, Tuscany can take a leading role in developing and validating AI tools, exploring multi-omics integration, and advancing computational pathology.

For long-term sustainability, the regional system must remain flexible and scalable. Digital architecture should support interoperability, allow for future upgrades, and be regularly assessed in terms of performance, equity, and impact. This includes adopting quality assurance metrics, benchmarking against national and international best practices, and engaging with global networks to remain aligned with evolving standards.

Finally, political and institutional support will be critical. Policymakers must back this transformation through clear regulation, dedicated funding, and incentives for inter-institutional collaboration.

By following this roadmap—anchored in infrastructure, innovation, education, governance, and sustainability—Tuscany can establish itself as a national and European reference model for digital pathology, accelerating its transition toward precision diagnostics and integrated, patient-centered care.

## Digital pathology: core requirements and implementation status in Tuscany

Tuscany has undertaken a progressive digital transformation of pathology services like a few other Italian regions, such as Veneto [33]. Nevertheless, nationwide DP implementation remains heterogeneous, hindered by infrastructural gaps, interoperability challenges, and an evolving regulatory framework.

To estimate the current state of DP readiness across Tuscany, a structured survey was conducted involving key stakeholders, including pathologists, IT specialists, and healthcare administrators from the TRHS. Each facility was asked to provide detailed information on annual diagnostic workload by slide type (Table 1), technologies in use, existing IT infrastructure, and human resources availability (Supplementary Tables 1, 2).

**Table 1:** Annual diagnostic workload by slide type. *H&E*, hematoxylin and eosin; *IHC*, immunohistochemistry; *LBC*, liquid-based cytology

Facilities	H&E (slides per year)	IHC/special stain (slides per year)	PAP-test/LBC (slides per year)	Total
AUSLTC	~ 260,000	~ 125,000	~ 50,000	~ 435,000
AUSLTNO	~ 420,000 (including ~ 2400 macroslides)	~ 116,000	~ 55,000	~ 591,000
AUSLSE	~ 180,000	~ 45,000	~ 12,000	~ 237,000
AOUC	~ 200,000	~ 85,000	~ 8,000	~ 293,000
AOUM	~ 5000	~ 3500	~ 250	~ 8750
AOUP	~ 260,000 (including ~ 7,500 macroslides)	~ 115,000	~ 70,000	~ 445,000
AOUS	~ 290,000	~ 67,000	~ 22,000	~ 379,000
TOTAL	~ 1,615,000	~ 556,500	~ 217,250	~ 2,388,750

### Image acquisition and scanning systems

The transition to DP requires investment in high-resolution slide scanners capable of handling various specimen types, including histological sections, cytology preparations, and frozen section slides [27]. Multiple scanner models are now available for routine use, each with specific advantages and limitations, making the choice of the most appropriate solution challenging [34]. Moreover, to ensure interoperability, scalability, and compliance with international standards, the adoption of the DICOM (Digital Imaging and Communications in Medicine) format for WSI is increasingly encouraged since it is recognized as the international reference (ISO 12052:2017). Originally developed for radiology—where it is mandatory as the only accepted format for archiving and exchange within RIS/PACS (Radiology Information Systems/Picture Archiving and Communication Systems) and national electronic health records—the format has since been extended to pathology by DICOM Working Group 26 [35]. Although not yet legally required in DP, it is explicitly referenced in Italian digital health strategies, such as the Electronic Health Record 2.0 (Fascicolo Sanitario Elettronico 2.0) [36], making it a *de facto *prerequisite for interoperability and long-term integration. By enabling structured metadata, standardized image encoding, and seamless PACS connectivity, DICOM supports cross-institutional exchange, durable archiving, and integration with AI tools, serving as a cornerstone for networked DP.

One of the key outcomes of the survey was a comprehensive mapping of slide scanning capabilities across the region. Although all institutions are equipped with at least one slide scanner, performance and features remain highly heterogeneous (Supplementary Table 1). With the reorganization of several pathology services into a hub-and-spoke model, case volumes and daily slide production have increased, concentrating high-throughput scanners in hub institutions and academic laboratories, where they can process hundreds of slides per day. Medium- and low-throughput scanners, by contrast, are mainly installed in spoke hospitals and are often used for specific purposes such as remote intraoperative examinations. Advanced functionalities (macroslide support, Z-stacking, and DICOM compatibility) and live streaming are available only for some models. Overall, this landscape highlights that, despite a broadly distributed technological base, the current equipment is still insufficient to support large-scale, routine digitalization. Expanding the number of high-throughput scanners and harmonizing available functionalities will be essential to achieve full DP implementation across the regional network.

### Workstations and viewing software

As part of the ongoing evaluation aimed at selecting the most suitable workstation for routine practice, several technical and ergonomic criteria must be considered. The ideal workstation should consist of one computer and two monitors, one for LIS and the other for WSI viewer. Monitors with high-pixel resolution and accurate color calibration are essential to assess images with the same diagnostic confidence as traditional glass slides. High-quality or medical-grade displays are therefore a pivotal component of the DP workflow: choosing the right monitor is as critical as selecting the most appropriate microscope [37–39]. In this regard, workstations equipped with CE-MDR certified medical displays (e.g., 4 K/8MP monitors) are recommended to ensure regulatory compliance (European Medical Device Regulation 2017/745), optimized color rendering, and consistent performance across laboratories. Beyond resolution, key specifications include high dots-per-inch (DPI) for crisp visualization of cellular and subcellular structures, wide color gamut coverage (e.g., AdobeRGB or DCI-P3) to faithfully reproduce histological stains, and uniform luminance to prevent perceptual inconsistencies. The use of ICC (International Color Consortium) profiles ensures standardized color rendering across acquisition, processing, and display systems. These parameters can have a significant impact on intra- and inter-observer concordance, particularly in diagnostically subtle or ambiguous cases. In parallel, vendor-provided viewing software, beyond simple visualization, should integrate real-time AI tools for automated measurements, quantitative scoring, and predictive analytics, further enhancing diagnostic capabilities [40].

In Tuscany, the evaluation process is already underway: some facilities have installed DP workstations, while others are testing different solutions to identify a shared standard for future deployment (Supplementary Table 2). Meanwhile, a regional assessment of hardware requirements is being conducted to estimate needs for the next 4 years, supporting the progressive scaling of DP infrastructure. This process is coordinated with LIS integration efforts to ensure full compatibility and usability across institutions, with the ultimate goal of deploying validated, ergonomic, and diagnostically reliable workstations across the regional DP network.

### Laboratory information systems (LIS) and workflow management

A LIS-based DP workflow supports automated sample logging, barcode tracking, and slide labeling, significantly reducing the risk of human error. Workflow automation also enhances laboratory efficiency by enabling smart case assignment based on pathologist expertise and workload balancing [40]. The cloud-enabled environment further supports second opinions, multidisciplinary tumor board reviews, and external consultations, making the system highly collaborative and network-aware. The implementation of real-time data analytics within the LIS allows laboratories to monitor key performance indicators such as TAT, case volume, and diagnostic concordance, supporting quality assurance and operational benchmarking [8]. Looking ahead, LIS platforms are expected to evolve into multimodal diagnostic cockpits, integrating clinical data with pathology workflows and embedding advanced analytics powered by AI and deep learning algorithms. These developments will further support diagnostic accuracy, predictive modeling, and personalized care, transforming the LIS from a tracking system into an active diagnostic partner for pathologists [16].

One of the key pillars of the Tuscany DP project is the standardization of diagnostic workflows across all pathology laboratories, aimed at building a fully integrated regional digital infrastructure. To achieve this, acquiring a uniform LIS was essential for managing every phase of the pathology workflow. The WINSAP 3.0 platform (Engineering®) was selected as the regional one; the rollout began in 2020 and is now adopted region-wide. Deployed in a cloud-based architecture, it ensures centralized management, seamless maintenance, and secure access via SPID (Sistema Pubblico di Identità Digitale). The system is integrated with the regional registry office and, in some hospitals, further connected to robotic storage systems, embedding and microtomy workstations, automatic stainers, and immunostainers, ensuring continuous process tracking and reducing the risk of patient misidentification. The platform also supports digital order entry, enabling electronic test requests, specimen traceability, and data synchronization with the DP infrastructure. However, a fully paperless workflow is not yet feasible, as printed documentation still serves as a legal and operational backup during specific workflow phases. The regional strategy therefore adopts a hybrid digital–paper model, ensuring interoperability, traceability, and compliance with current regulatory requirements while progressively transitioning toward full digitalization. Furthermore, the platform provides the foundation for structured and interoperable digital reporting, implemented through LIS-based templates, checklists, and controlled terminologies (such as SNOMED CT, ICD-O, and LOINC) aligned with national and international standards, to ensure semantic consistency and data interoperability. WINSAP 3.0 currently supports HL7-based data exchange, while DICOM/DICOM-SR compliance will be managed within the DP image layer. This architecture will enable standardized, machine-readable diagnostic reports and ensure readiness for AI-assisted analytics and registry harmonization across the regional network.

Although WINSAP 3.0 is technically DP-ready, full LIS–DP integration within the TRHS remains limited. Apart from Carrara Hospital, where integration is partially operational, the platform is not yet interfaced with DP workflows. Full workflow integration, including remote access, digital report signing, and AI-assisted diagnostics, is not yet implemented. In addition, no institution has formally assigned personnel dedicated to coordinating DP activities at this stage (Supplementary Table 2). To address these gaps, ESTAR, in collaboration with the regional pathology network, has undertaken a series of preparatory activities to enable full LIS–DP integration. These include the design of multi-institutional integration scenarios, compiling of an updated inventory of DP devices to be interfaced, and definition of technical requirements for connectivity with existing Vendor Neutral Archives (VNAs). Prospective assessments have also been carried out to evaluate the impact of DP on data transmission and storage infrastructures, together with the updating of the catalogue of pathology-related applications. As part of this process, WINSAP 3.0 will be configured in a centralized, multitenant architecture, in which a single regional installation serves all pathology laboratories while ensuring secure separation of institutional data. This model streamlines maintenance, guarantees uniform updates, and facilitates interoperability across the TRHS.

Finally, a shared approval process has been established for telepathology use cases, structured at three levels: intra-facility (within hospitals of each macro-area), inter-facility in a hub-and-spoke model (linking local hospitals to regional referral centers), and national/international collaboration (with dedicated reference centers). A LIS-integrated module will enable digital report signing, case tracking, access to shared patient history (with consent), and real-time communication among pathologists. Initial applications have been prioritized in high-impact domains, including dermatopathology, breast pathology, hematopathology, neuropathology, uropathology, and transplant diagnostics. At this stage, routine cytology digitization is not a program priority; however, pilot initiatives on liquid-based cytology and selected diagnostic smears are underway to evaluate technical feasibility, multi-focus (z-stack) acquisition, and integration into existing digital workflows.

### Connectivity, data storage, and management

DP generates massive data volumes, encompassing WSI, structured clinical information, and metadata, essential for analysis and traceability. This creates a pressing need for robust, scalable, and secure storage infrastructures. An essential decision concerns whether to adopt cloud-based, on-premises, or hybrid solutions, each with specific trade-offs in terms of cost, accessibility, scalability, and regulatory compliance. Cloud infrastructures offer flexibility, scalability, and cross-institutional accessibility, whereas local servers provide more direct control over data governance, latency, and institutional policies. Operationally, a hybrid model is often considered optimal: cloud servers act as centralized long-term archives accessible to all regional pathology labs, while local servers function as short-term, high-speed caches supporting daily diagnostic workflows.

**Fig. 2:**
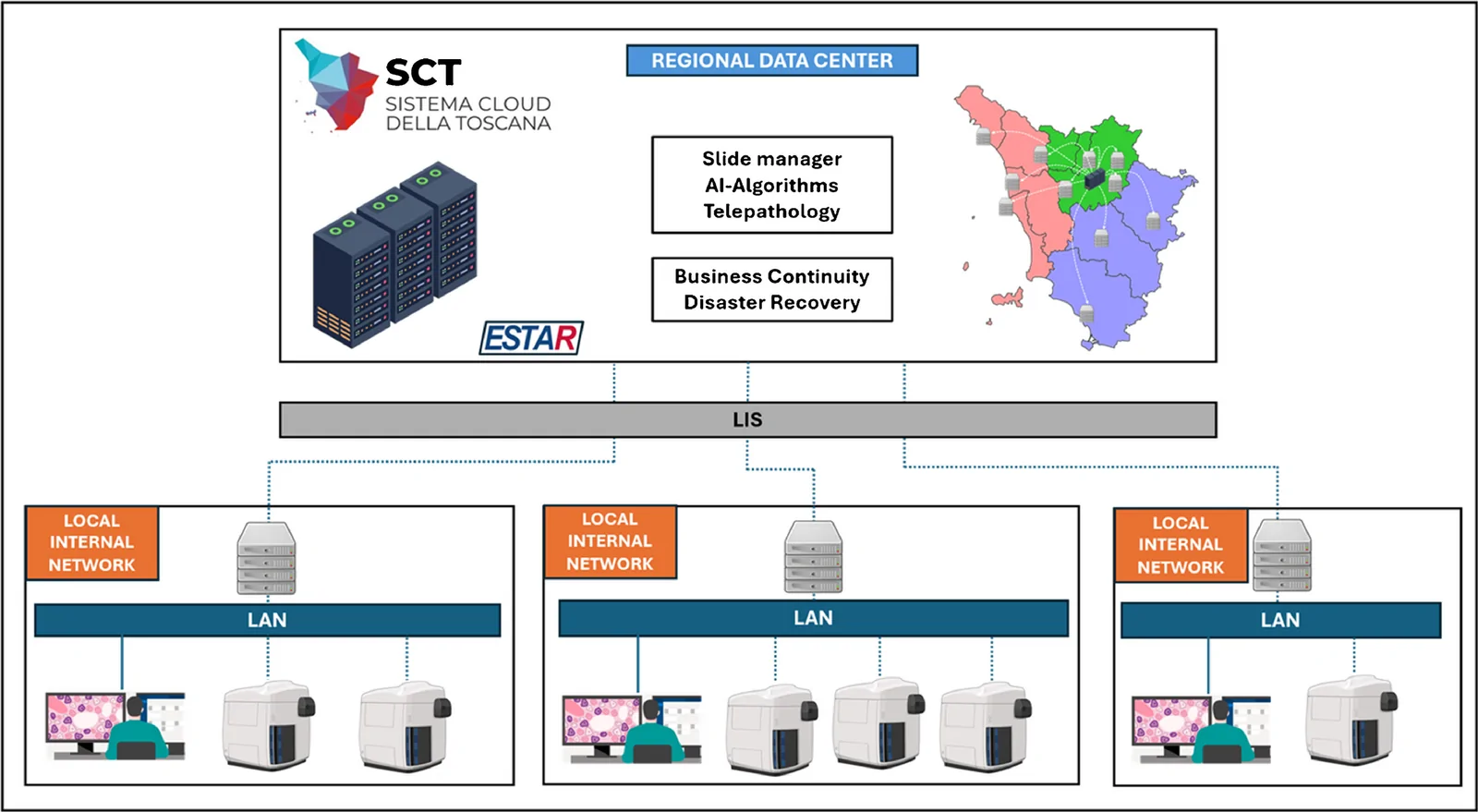
Architecture of the potential regional digital pathology infrastructure in Tuscany. The system is based on a centralized model coordinated through the SCT (Sistema Cloud della Toscana) data center, managed by ESTAR. The architecture enables shared services such as slide management, AI-based image analysis, and telepathology, with integrated business continuity and disaster recovery functions. Each local hospital operates scanners and workstations within its internal network, connected to a centralized laboratory information system (LIS) and interfacing with the regional cloud by middleware servers for data exchange and coordination

The Tuscany DP project adopts a hybrid model in which daily operations are supported by the Tuscany Cloud System (SCT), a public, cloud-compliant, centralized infrastructure located in Florence that hosts DP applications and database servers, while local middleware servers manage connections with scanners. Business continuity and disaster recovery are ensured through SCT, whereas local servers interface the cloud with institutional equipment (Fig. 2). Integration into SCT implicates upgrading legacy pathology software for cloud compatibility, harmonizing and cleaning local databases, and migrating them into a centralized cloud-based instance. In parallel, the region is advancing interoperability between DP platforms, LIS, and the wider healthcare network, while deploying a centralized viewer for diagnostic imaging.

In terms of connectivity, all regional hospitals are currently provisioned with 1 Gbps inter-hospital connections, while pathology departments report symmetrical 100/100 Mbps bandwidth at the workstation level, a configuration generally sufficient for real-time slide review and well-suited to support scalable DP operations and centralized data exchange, and broadly consistent with international recommendations [41–45]. However, to sustain high-throughput workloads such as continuous WSI acquisition, multi-user access, and telepathology, further upgrades are needed; priorities include: (i) establishing secure VPN access to guarantee encrypted and compliant inter-facility data transfer; (ii) implementing quality of service (QoS) protocols to prioritize diagnostic traffic and minimize latency during peak use; and (iii) adopting efficient image streaming technologies to optimize remote visualization without compromising quality. The regional plan foresees strengthening and expanding the regional network infrastructure to provide robust, resilient connections among all Tuscan hospitals and to support future DP growth.

Finally, another unsolved issue worldwide is the scope of digital archiving: should every scanned slide be preserved, or only those selected by the reporting pathologist? This has implications for both storage capacity and legal accountability. An even more pressing and controversial challenge concerns the retention time for DP data. Current guidelines, such as those from the College of American Pathologists (CAP) and the Royal College of Pathologists (RCPath), recommend storing glass slides for a minimum of 10 years and digital images for several years, though exact durations vary [45–48]. At present, no specific regulatory framework exists for WSI retention, either at the international or national level, and no universally accepted standards have yet been defined. In this regulatory vacuum, pathology departments and regional health authorities are responsible for defining context-appropriate retention policies [40]. Moreover, as AI becomes increasingly integrated into pathology practice, the need for structured, high-quality digital repositories grows. Such archives will be pivotal in supporting the training, validation, and benchmarking of machine learning algorithms, underscoring the strategic importance of long-term data stewardship in DP.

### Artificial intelligence integration

Within the TRHS, the progressive implementation of DP across all pathology facilities provides the foundation for integrating AI-driven diagnostic tools. Deep learning algorithms trained on large, annotated histopathology datasets can assist pathologists in detecting subtle morphological changes, classifying tumor subtypes, and predicting clinical outcomes from tissue architecture and biomarker expression patterns. Embedded in the regional digital infrastructure, AI solutions could support case triage, highlight regions of interest, and generate preliminary or differential diagnoses. These functionalities have the potential to reduce interobserver variability and streamline workflows, particularly in high-volume centers and for second-opinion consultations across the network [16].

Several CE-IVD certified AI solutions are already available on the market and could be progressively adopted to support pathology workloads across regional hospitals. However, successful implementation will require rigorous clinical validation, regulatory compliance, and dedicated training pathways for pathology professionals. Standardization of training datasets, bias mitigation strategies, and continuous monitoring of algorithm performance will also be crucial to ensure safe and effective use across diverse patient populations and institutions.

At the current stage of the project, AI algorithms are not yet implemented in routine practice but have been identified for structured evaluation through regional pilot projects focused on diagnostic support, biomarker quantification, and quality assurance. The implementation plan will define criteria for validation, interoperability, and regulatory compliance. Leveraging its harmonized digital infrastructure and centralized governance, Tuscany provides a controlled framework for the future introduction of AI-assisted pathology.

## Overcoming barriers and challenges: the Tuscan approach

Despite the advances made in the implementation of DP across Tuscany, several challenges still limit its full integration into routine clinical practice.

Technical heterogeneity among scanners, file formats, and software platforms may hinder interoperability; thus, the deployment of a centralized, multi-format viewer is currently underway and is expected to obtain full certification by the end of the year [4, 38–40].

Infrastructure remains uneven. Although sufficient inter-hospital connectivity and workstation bandwidth are available, the lack of VPN access and limited storage volume may reduce performance and interoperability. To address this, the region has adopted a hybrid architecture with planned bandwidth upgrades and secure access protocols [27].

Financial and logistical limitations, particularly in spoke centers, have slowed adoption due to limited budgets, staffing, and strategic planning capacity. However, Tuscany has secured funding through the PNRR-Mission 6, with ESTAR coordinating centralized purchasing and implementation. Official data will be released upon completion of the current PNRR M6C2 I1.1.1 framework (2025–2026), marking the successful conclusion of the first phase of infrastructure modernization. Subsequent developments will be supported through complementary regional and institutional funding, ensuring progressive expansion and long-term sustainability of the DP network.

Compared with other Italian scenarios, where DP adoption remains largely fragmented and hospital-driven, Tuscany has developed one of the few regionally coordinated frameworks for digital transformation. Supported by ESTAR’s centralized governance, a shared LIS (WINSAP 3.0), and unified procurement processes, the region will benefit from workflow standardization, interoperability, and cost efficiency across pathology units. These structural advantages have accelerated digital integration compared with the national average. Nevertheless, several systemic challenges persist, including heterogeneous digital maturity among institutions, complex administrative procedures, and the need for continuous training and change management, issues that mirror those faced across the broader Italian healthcare landscape.

Regulatory uncertainties, particularly regarding slide retention policies and legal accountability in AI-assisted diagnostics, continue to create hesitation. To mitigate these issues, a phased and validated AI rollout is planned in alignment with evolving EU regulations and GDPR compliance [19–22]. Following approval by clinical stakeholders, the document outlining the proposed use cases was submitted and provisionally approved by the Data Protection Officers (DPO) Board. The document was then integrated with a formal risk analysis and a compliance checklist addressing security and privacy requirements. Final evaluation by the DPO Board is currently pending.

Culturally, some resistance persists among pathologists, especially in centers without training or early exposure to DP. The regional strategy addresses this through stepwise adoption in selected subspecialties and structured training programs.

Looking ahead, the infrastructure is designed to be scalable, modular, and interoperable, ensuring readiness for future developments such as AI integration and multi-omics diagnostics.

Overcoming these challenges requires a coordinated, multi-level strategy involving regional governance, institutional leadership, IT services, and continuous professional development. Thanks to its strong infrastructure and unified healthcare governance, Tuscany is well positioned to meet these challenges through targeted investments and a shared regional vision.

## Conclusion

DP implementation in TRHS marks a strategic shift toward more accurate, efficient, and connected diagnostics. A phased, coordinated approach—based on shared infrastructure, standard protocols, and continuous training—can overcome existing barriers and enable full clinical integration.

The establishment of a regional DP network, supported by AI and cloud-based systems, lays the groundwork for innovation in diagnostics, education, and research. With appropriate investment and regulatory alignment, Tuscany is well positioned to become a national reference for DP, advancing precision medicine and sustainable healthcare.

## Supplementary Information

Below is the link to the electronic supplementary material.

ESM 1 (DOCX 17.5 KB)

## Data Availability

Original data are available from the corresponding author upon reasonable request.
