# Supplementary material for: Toward a regional digital pathology network in Tuscany: current status and implementation roadmap
Source: Virchows Arch. 2026 Jan 28;489(4):973–83. doi: 10.1007/s00428-026-04408-2 (PMC13714043; doi:10.1007/s00428-026-04408-2)
Supplement: Supplementary file 1 [file 428_2026_4408_MOESM1_ESM.docx]

**Supplementary Table 1**. Slide Scanning Hardware and Technical Capabilities. ^§^Used for research purposes.

| **Facilities** | **Slide Scanners** | **Throughput** | **Objective Lens** | **Macroslides** | **Z-stack** | **Fluorescence** | **DICOM** | **Live Streaming** |
| --- | --- | --- | --- | --- | --- | --- | --- | --- |
| AUSLTC | 6× Menarini DEEPINTO  (Pescia, Pistoia, Torregalli, Santa Maria Annunziata, Empoli, Prato Hospitals) | Low | 4x, 10x, 20x, 40x | NO | NO | NO | NO | YES |
| AUSLTNO | 1x Leica Aperio GT450  (Carrara Hospital) | High | 20x, 40x | NO | YES | NO | YES | NO |
|  | 5x Menarini DEEPINTO  (Carrara,  Massa, Versilia, Lucca, Livorno Hospitals) | Low | 4x, 10x, 20x, 40x | NO | NO | NO | NO | YES |
| AUSLSE | 1x Ventana DP200  (Grosseto Hospital) | Low | 20x, 40x | YES | YES | NO | YES | NO |
| AOUC / AOUM | 1x Leica Aperio GT450 | High | 20x, 40x | NO | YES | NO | YES | NO |
|  | 1x Aperio AT2^§^ | High | 20x, 40x | YES | YES | NO | YES | NO |
|  | 1xVisiaLab NaviLive | Low | 4x, 10x, 20x, 40x | NO | NO | NO | NO | YES |
| AOUP | 1x 3DHISTECH Pannoramic1000 | High | 20x, 40x | YES | YES | NO | YES | NO |
|  | 1x Aperio AT2^§^ | High | 20x, 40x | YES | YES | NO | YES | NO |
|  | 1x Ventana DP200 | Low | 20x, 40x | YES | YES | NO | YES | NO |
| AOUS | 1x Leica Aperio GT450 | High | 20x, 40x | NO | YES | NO | YES | NO |
|  | 1x Hamamatsu Nanozoomer SQ | Low | 20x, 40x | NO | YES | NO | NO | NO |
|  | 1x Ventana DP200 | Low | 20x, 40x | YES | YES | NO | YES | NO |

**Supplementary Table 2.** Laboratory Information Systems, Accessibility, Hardware, AI Integration, and Personnel Readiness. *LIS DP-ready but not integrated yet. ^#^LIS DP-ready but integration with Aperio Imagescope viewer only at Carrara and Lucca Hospitals. ^§^Mainly used for imaging analysis.

| **Facilities** | **LIS** | **LIS-DP Integration** | **Workstations in use** | **Remote Access to LIS** | **Remote Digital Sign-out** | **AI algorithms for routine sign-out** | **Dedicated personnel** |
| --- | --- | --- | --- | --- | --- | --- | --- |
| AUSLTC | WINSAP 3.0 | NO* | NO | NO | NO | NO | NO |
| AUSLTNO | WINSAP 3.0 | YES^#^ | 13x at Carrara Hospital  3x at Lucca Hospital  Dual monitor configuration:  1x Fujitsu P27-8T, 27”, QHD, 4MP, 60Hz, 8-bit  1x Hannspree HP270WJB, 27”, Full HD  CPU: AMD Ryzen 5 2600 (6-core 3.4 Ghz)  RAM: 16 GB GPU: Radeon RX550/550 Stoarge: 250 GB (SSD)  Network Bandwidth Up/Down: 1 Gbps | NO | NO | NO | NO |
| AUSLSE | WINSAP 3.0 | NO* | NO | NO | NO | NO | NO |
| AOUC | WINSAP 3.0 | NO* | 1x HP Z8^§^  Single monitor configuration:  1x Dell UP3216Q, 31.5”, UHD, 60Hz, 10-bit, AdobeRGB/DCI-P3  CPU: 2x Intel Xeon Gold 6132 (14 core 2.60GHz)  RAM: 112 GB  GPU: Integrated (40 core)  SSD: 10 TB  Network Bandwidth Up/Down: 1 Gbps | NO | NO | NO | NO |
| AOUM | WINSAP 3.0 | NO* | NO | NO | NO | NO | NO |
| AOUP | WINSAP 3.0 | NO* | 1x Mac mini^§^  Dual monitor configuration:  1x Apple Studio Display, 27”, 5K, 15MP, 60Hz, 10-bit, DCI-P3  1x Dell U2720Q, 27”, UHD, 8MP, 60Hz, 10-bit, DCI-P3  CPU: M4 Pro (14 core)  RAM: 64 GB  GPU: Integrated (20 core)  Storage: 1TB (SSD)  Network Bandwidth Up/Down: 100 Mbps | NO | NO | NO | NO |
|  |  |  | 1x iMac^§^  Dual monitor configuration:  1x Integrated Display, 24”, 4.5K, 11MP, 60Hz, 10-bit, DCI-P3  1x ThinkVision E24-29, 24”, Full HD  CPU: M1 (8 core)  RAM: 8 GB  GPU: Integrated (8 core)  Storage: 1TB (SSD)  Network Bandwidth Up/Down: 100 Mbps. |  |  |  |  |
|  |  |  | 1x Windows Workstation^§^  Dual monitor configuration:  2x Dell U2720Q, 27”, UHD, 8MP, 60Hz, 10-bit, DCI-P3  CPU: XEON Gold 5217 (8 core 3.0 Ghz)  RAM: 48 GB  GPU: NVIDIA RTX 5000 (16GB VRAM)  Storage: 1TB NVME + 12 TB HDD  Network Bandwidth Up/Down: 100 Mbps. |  |  |  |  |
|  |  |  | 1x slide manager installed on IHPE ProLiant ML110 Gen10 Server^§^  CPU: Intel Xeon Silver 4110 (8 core - 2.1 GHz)  RAM 16 GB  Storage: 2x 1TB (HDD in RAID 1); QNAP TS431P (24TB HDD in RAID 5)  Network Bandwidth Up/Down: 100 Mbps |  |  |  |  |
| AOUS | WINSAP 3.0 | NO* | NO | NO | NO | NO | NO |
